# Extended thromboprophylaxis after hip fracture surgery: Real-world evidence of direct oral anticoagulants versus low molecular weight heparin of unfractionated heparin

**DOI:** 10.1371/journal.pone.0343020

**Published:** 2026-03-12

**Authors:** María Manuela Clavijo, Juan Ignacio Ruiz, Martina Rodriguez Brindicci, Paula Rodriguez, Victoria Wauters, María de los Angeles Vicente Reparaz, Alex Mountford, Micaela Monteagudo, Manuela Camarero, Claudia Erica Casali, Carolina Mahuad, Florencia Aizpurua, Marta Elisa Zerga, Adriana Ventura, Nicolas Siciliano, Gonzalo Garate

**Affiliations:** 1 Department of Hematology, Hospital Alemán, Ciudad Autónoma de Buenos Aires, Argentina; 2 Department of Clinical Medicine, Hospital Alemán, Ciudad Autónoma de Buenos Aires, Argentina; Celal Bayar University: Manisa Celal Bayar Universitesi, TÜRKIYE

## Abstract

**Background:**

Hip fracture surgery carries the highest postoperative risk of venous thromboembolism (VTE) among surgical populations. Guidelines recommend low molecular weight heparin (LMWH) over direct oral anticoagulants (DOACs) due to limited evidence. However, oral administration and cost considerations have led to widespread real-world use of DOACs. This study aimed to compare the effectiveness and safety of extended thromboprophylaxis with DOACs versus LMWH or unfractionated heparin (UFH) after hip fracture surgery.

**Methods:**

We conducted a retrospective cohort study of adults undergoing hip fracture surgery at Hospital Alemán, Buenos Aires, Argentina (January 2011–June 2025), covered by the hospital’s healthcare insurance. Eligible patients were discharged with extended pharmacologic thromboprophylaxis (LMWH, UFH, or DOACs). Outcomes within 3 months included VTE, major or clinically relevant non-major bleeding (MB/CRNMB), and all-cause mortality. Multivariable Cox regression, Fine-Gray models, and propensity score adjustments were applied.

**Results:**

Of 425 hip fractures, 340 cases (301 patients) met eligibility criteria. Extended prophylaxis used was LMWH/UFH in 102 cases and DOACs in 238. VTE occurred in 2.5% of DOAC and 3.9% of LMWH/UFH cases. MB/CRNMB occurred in 2.1% vs 5.9%, and mortality in 3.0% vs 3.9%, respectively. Adjusted hazard ratios for DOACs versus LMWH/UFH were 0.64 (95% CI, 0.16–2.49) for VTE, 0.69 (0.15–2.16) for bleeding, and 0.73 (0.22–2.37) for mortality.

**Conclusions:**

In this real-world cohort, DOACs showed comparable effectiveness and safety to LMWH/UFH for extended prophylaxis after hip fracture surgery. These findings support DOACs as a potential alternative in this high-risk population, pending confirmation in prospective studies.

## Introduction

Hip fractures are a common and growing health problem worldwide, particularly among older adults, and are associated with substantial morbidity and mortality [1–3]. Among the complications that follow hip fracture surgery, venous thromboembolism (VTE) remains one of the most serious and potentially life-threatening events [4].

Patients undergoing orthopedic surgery face the highest risk of VTE compared with other surgical populations [5]. Before the routine use of pharmacologic thromboprophylaxis, clinical trials reported postoperative (POP) VTE rates of 15–30% [5].The introduction of systematic anticoagulant prophylaxis has reduced this risk to approximately 1–5% [5]. Nevertheless, in the specific context of hip fracture surgery, postoperative VTE incidence remains both higher and markedly heterogeneous, with studies reporting rates as high as 20% [6–8] and, in some cases, exceeding 50% [1,9].

Since the advent of direct oral anticoagulants (DOACs), numerous observational studies have explored their use after hip fracture surgery [10–16]. Collectively, these investigations suggest that DOACs may offer a feasible therapeutic alternative; however, the evidence is limited by small sample sizes and considerable imprecision. Current international guidelines, including those of the American Society of Hematology and the American College of Chest Physicians, recommend low molecular weight heparin (LMWH) over DOACs in this setting, citing the lack of robust supporting data [5,17]. Despite these recommendations, DOACs are increasingly prescribed in everyday clinical practice, driven by their ease of oral administration, lower costs, and the extrapolation of efficacy data from elective orthopedic surgery.

In this study, we aimed to evaluate the comparative effectiveness and safety of extended thromboprophylaxis with DOACs versus LMWH or unfractionated heparin (UFH) in patients undergoing hip fracture surgery in a real-world clinical setting.

## Materials and methods

We conducted a retrospective cohort study using electronic health records from the Hospital Alemán healthcare plan insurance in Buenos Aires, Argentina. Access to the data for research purposes began on February 16, 2024. All authors are physicians working at the same institution, actively involved in patient care, and therefore have authorized access to the institutional system using individual professional identification. The data were accessed and analyzed in accordance with institutional ethical standards and regulatory requirements. No information that could directly identify individual participants was accessed or disclosed at any stage during or after data collection.

The study included adults (≥18 years) who underwent hip fracture surgery between January 2011 and June 2025 and were discharged with extended pharmacologic thromboprophylaxis. Patients were excluded if they had a diagnosis of venous thromboembolism (VTE) during the in-hospital period after hip fracture, an indication for therapeutic anticoagulation, or incomplete clinical data. Extended prophylaxis consisted of low-molecular-weight heparin (LMWH), unfractionated heparin (UFH), or direct oral anticoagulants (DOACs; rivaroxaban, apixaban, or dabigatran). According to standard clinical practice at our center, enoxaparin was the LMWH used for thromboprophylaxis. Anticoagulant regimens were as follows: enoxaparin 40 mg once daily (increased to 60 mg once daily in patients with a body mass index ≥30 kg/m² and reduced to 30 mg once daily in those with a creatinine clearance [CrCl] <30 mL/min), unfractionated heparin (UFH) 5,000 IU twice daily, rivaroxaban 10 mg once daily, apixaban 2.5 mg twice daily, and dabigatran 220 mg once daily. Dabigatran was not prescribed to patients with a CrCl < 60 mL/min or to those older than 70 years. In patients with a CrCl < 30 mL/min, apixaban was the only direct oral anticoagulant used after hospital discharge. No dose adjustments were applied for anticoagulants other than enoxaparin.

The primary outcome was VTE, defined as objectively confirmed deep vein thrombosis (DVT) or pulmonary embolism (PE) within 3 months after discharge. Secondary outcomes included a composite of major bleeding (MB) or clinically relevant non-major bleeding (CRNMB), and all-cause mortality. Bleeding was defined according to the ISTH classification as: a) MB: hemoglobin drop of >2 g/ dL, transfusion of >2 units of packed red blood cells, symptomatic bleed in a critical area (intracranial, retroperitoneal, intraspinal, intraocular), or fatal bleed; b) CRNMB: one which requires or prolongs hospitalization, results in laboratory testing, imaging, compression, a procedure, interruption of the study medication, or a change in concomitant therapies [18]. Time-to-event outcomes were assessed using Kaplan-Meier survival curves with log-rank tests, while multivariable Cox proportional hazards models were applied to adjust for potential confounders. Fine–Gray competing risk models were used to account for death as a competing event, and propensity score methods were employed to adjust for baseline differences across treatment groups. Analyses also accounted for clustering since there were patients who underwent more than one hip fracture surgery during the study period. For statistical analysis, Rstudio version 2023.06.0 + 421 was used.

## Statement of ethics

This study protocol was reviewed and approved by Comité de ética independiente del Hospital Alemán (CEHIA), Av Pueyrredon 1640, Buenos Aires, Argentina. Tel: + 54 11 4827-7000. Informed consent was not obtained. Resolution 1480/2011 of the National Ministry of Health exempts retrospective cohort studies from the obligation to obtain informed consent. The confidentiality of each participant’s identity was adequately preserved according to local regulations (Administración Nacional de Medicamentos, Alimentos y Tecnología [ANMAT]).

## Results

**Fig 1:**
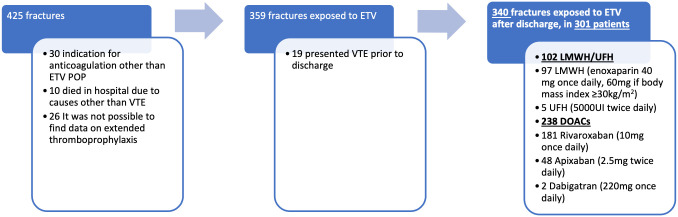
Study Population Flowchart: eligibility criteria and thromboprophylaxis exposure.

**Fig 2:**
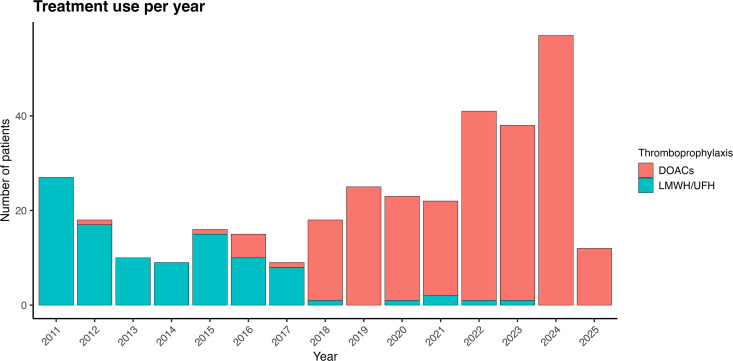
Annual Trends in Extended Thromboprophylaxis Use After Hip Fracture Surgery (2011–2025).

A total of 425 hip fractures were identified, of which 340 cases (301 patients) were eligible (Fig 1). Median age was 83 years (interquartile range [IQR] 77.3–88.4), with 81% female. The characteristics of the population analyzed overall and by treatment received are described in Table 1. All patients received either UFH or LMWH during hospitalization for hip fracture. In 10 of 340 cases (2.9%), the thromboprophylaxis strategy consisted of UFH, while the remaining patients received LMWH (enoxaparin). Thromboprophylaxis was initiated within 24 hours of hospital admission and was discontinued 12–24 hours before surgery. Postoperatively, the median time to treatment initiation was 15 hours (IQR12–23) in the overall population, 13 hours in the LMWH/UFH group (IQR 12–24), and 15 hours in the DOAC group (IQR 12–22), p = 0.14. Extended prophylaxis was prescribed for one month duration after discharge. It included LMWH/UFH in 102 cases (97 LMWH, 5 UFH) and DOACs in 238 cases (181 rivaroxaban, 48 apixaban, 2 dabigatran), Fig 1. DOACs use predominated from 2018 onwards (Fig 2).

**Table 1:** Baseline characteristics.

*Variable*	Category	Total (N = 340)	LMWH/UFH (N = 102)	DOACs (N = 238)	p-value
***Sex (%)***	Female	274 (80.6)	81 (82.7)	190 (79.8)	0.697
	Male	66 (19.4)	17 (17.3)	48 (20.2)	
***Fracture (%)***	Medial	207 (60.9)	68 (66.7)	139 (58.4)	0.012
	Lateral	98 (28.8)	19 (18.6)	79 (33.2)	
	Other/Unknown	35 (10.3)	15 (14.7)	20 (8.4)	
***Surgery (%)***	Total arthroplasty	144 (42.5)	55 (53.9)	89 (37.6)	0.008
	Partial arthroplasty	90 (26.5)	26 (25.5)	64 (27.0)	
	Osteosynthesis	105 (31.0)	21 (20.6)	84 (35.4)	
***Synchronous fracture (%)***	Yes	27 (7.9)	11 (10.8)	16 (6.7)	0.204
	No	313 (92.1)	91 (89.2)	222 (93.3)	
***Anesthesia (%)***	General	103 (30.3)	18 (17.6)	85 (35.7)	0.001
	Block + sedation	228 (67.1)	79 (77.5)	149 (62.6)	
	Other	9 (2.6)	5 (4.9)	4 (1.7)	
***ASA score (%)***	<3	195 (57.7)	65 (65.0)	130 (54.6)	0.079
	≥3	143 (42.3)	35 (35.0)	108 (45.4)	
***History of VTE (%)***	Yes	10 (2.9)	2 (2.0)	8 (3.4)	0.484
	No	330 (97.1)	100 (98.0)	230 (96.6)	
***Known thrombophilia (%)***	Yes	1 (0.3)	0 (0.0)	1 (0.4)	0.512
	No	339 (99.7)	102 (100.0)	237 (99.6)	
***COVID-19 during follow-up (%)***	Yes	6 (1.8)	0 (0.0)	6 (2.5)	0.104
	No	334 (98.2)	102 (100.0)	232 (97.5)	
***Active cancer (%)***	Yes	34 (10.0)	9 (8.8)	25 (10.5)	0.637
	No	306 (90.0)	93 (91.2)	213 (89.5)	
***Smoking (%)***	Active	42 (12.4)	16 (15.7)	26 (10.9)	0.221
	No active smoking	298 (87.6)	86 (84.3)	212 (89.1)	
***COPD (%)***	Yes	34 (10.0)	14 (13.7)	20 (8.4)	0.134
	No	306 (90.0)	88 (86.3)	218 (91.6)	
***Autoimmune disease (%)***	Yes	22 (6.5)	5 (5.0)	17 (7.1)	0.611
	No	317 (93.5)	96 (95.0)	221 (92.9)	
***Hypertension (%)***	Yes	220 (64.7)	62 (60.8)	158 (66.4)	0.322
	No	120 (35.3)	40 (39.2)	90 (33.6)	
***Diabetes mellitus (%)***	Yes	39 (11.5)	12 (11.8)	27 (11.3)	0.911
	No	301 (88.5)	90 (88.2)	211 (88.7)	
***Dyslipidemia (%)***	Yes	73 (21.5)	26 (25.5)	47 (19.8)	0.245
	No	266 (78.5)	76 (74.5)	190 (80.2)	
***Coronary artery disease (%)***	Yes	31 (9.1)	10 (9.8)	21 (8.8)	0.774
	No	309 (90.9)	92 (90.2)	217 (91.2)	
***Stroke (%)***	Yes	25 (7.4)	8 (7.8)	17 (7.1)	0.821
	No	315 (92.6)	94 (92.2)	221 (92.9)	
***Peripheral artery disease (%)***	Yes	37 (10.9)	12 (11.8)	25 (10.5)	0.732
	No	303 (89.1)	90 (88.2)	213 (89.5)	
***Period (%)***	2011–2017	103 (30.3)	96 (94.1)	7 (2.9)	<0.001
	2018–2025	237 (69.7)	6 (5.9)	231 (97.1)	
***Age: median (IQR)***	–	83.29 (77.27–88.36)	81.65 (77.67–88.99)	83.76 (77.67–87.12)	0.085
***Surgery time (min), mean (SD)***	–	90.05 (33.58)	99.47 (32.62)	86.09 (33.26)	<0.001
***Creatinine clearance, median (IQR)***	–	72.0 (57.0–86.0)	79.0 (59.5–89.0)	71.0 (56.0–83.0)	0.075
***Days to surgery, median (IQR)***	–	3 (2 –4)	2.5 (2 –3)	3 (2 –4)	0.071
***Hospital stay (days), median (IQR)***	–	9 (7 –12)	9 (8 –12)	9 (7 –12)	0.995
***BMI, median (IQR)***	–	24.34 (21.51–27.49)	24.31 (21.52–26.92)	24.39 (21.52–27.62)	0.519

LMWH = Low Molecular Weight Heparin; UFH = Unfractionated Heparin; DOACs = Direct Oral Anticoagulants; VTE = Venous Thromboembolism; COPD = Chronic Obstructive Pulmonary Disease; ASA = American Society of Anesthesiologists; BMI = Body Mass Index; SD = Standard Deviation; IQR = Interquartile Range.

During the 3-month follow-up, VTE occurred in 10 patients (2.9% overall): 6 cases in the DOAC group (2.5%) and 4 in the LMWH/UFH group (3.9%). Regarding the subtype of event, there were 6 cases of PE, 2 of distal DVT, and 2 of proximal DVT. All cases were symptomatic; the diagnosis of PE was established by CT scan, and DVT was diagnosed by Doppler ultrasonography. One PE event was fatal. It occurred in the LMWH/UFH group in an 81-year-old woman with COPD who developed bilateral pulmonary embolism involving the main pulmonary arteries. She was admitted five days after hospital discharge and died four days later due to respiratory failure. Among the 10 patients who developed VTE, the median age was 84.35 years (IQR 81.74–86.18), all were female, 7 patients had medial fractures, 7 underwent partial arthroplasty, 2 osteosynthesis, and 1 total arthroplasty. Regarding anesthesia, 9 patients received regional block with sedation, 6 patients had an ASA score ≥3. Comorbidities included chronic obstructive pulmonary disease in 3 patients, active cancer in 1, and current smoking in 2. Time to thromboprophylaxis initiation was not associated with VTE (HR 0.99, 95% CI 0.96–1.01) and was unrelated to time to surgery (p = 0.187) or anesthesia type (p = 0.710). Patient characteristics are detailed in the Supplementary Material (S1 Table).

Regarding secondary outcomes, MB/CRNMB was observed in 12 patients (3.5% overall), including 6 cases with DOACs (2.5%) and 6 with LMWH/UFH (5.9%). Among the 12 patients who experienced bleeding complications, 7 events were classified as major bleeding. These included 3 cases of gastrointestinal bleeding (2 occurring in patients receiving LMWH and 1 fatal event in a patient treated with rivaroxaban) and 4 cases of surgical-site bleeding (1 associated with apixaban, 1 with rivaroxaban, and 2 with LMWH). The fatal event occurred in a 91-year-old woman with severe aortic stenosis and a history of mucosal bleeding, including epistaxis, oral, and gastrointestinal bleeding. The remaining 5 events were classified as clinically relevant non-major bleeding (CRNMB), comprising 4 surgical-site hematomas (2 with LMWH, 1 with rivaroxaban, and 1 with apixaban) and 1 case of gastrointestinal bleeding in a patient receiving rivaroxaban. Patient characteristics are reported in the Supplementary Material (S2 Table).

**Fig 3:**
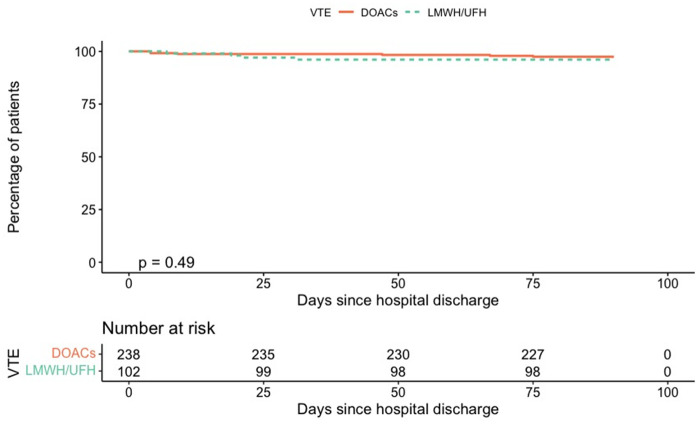
Kaplan–Meier Curve for Post-Discharge VTE Incidence by Thromboprophylaxis Type.

**Fig 4:**
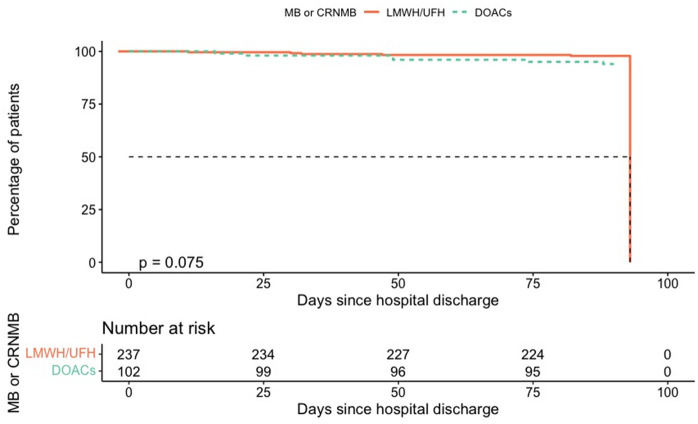
Kaplan–Meier Curve for Post-Discharge MB/CRNMB Incidence by Thromboprophylaxis Type.

A total of 11 deaths were recorded (3.2% overall): 7 patients in the DOAC group (3.0%) and 4 in the LMWH/UFH group (3.9%). Of the 11 deaths recorded, 6 were attributed to respiratory infection (4 of these patients underwent computed tomography scanning that ruled out pulmonary embolism), 1 to pulmonary embolism, 1 to gastrointestinal bleeding, and 1 to cardiac failure; in 2 cases, the cause of death was not reported. Patient characteristics are reported in the Supplementary Material (S3 Table). Kapplan-Meier curves for time to VTE and MB/CRNMB are shown in Figs 3 and 4.

The incidence of VTE, MB/CRNMB, and mortality stratified by clinical characteristics such as age, creatinine clearance, ASA score, fracture type, and type of surgery is shown in Supplementary S4 Table.

In multivariable Cox and Fine–Gray regression analyses, prior VTE was the only variable independently associated with VTE occurrence (hazard ratio [HR] 8.87, 95% CI 2.13–36.98, sub hazard ratio [SHR] 8.89, 97.5% CI 2.01–39.35), whereas thromboprophylaxis with DOACs was not significantly different from LMWH/UFH, although a nonsignificant reduction in VTE risk was observed (HR 0.64, 95% CI 0.16–2.49, SHR 0.62, 95% CI 0.16–2.40) (Table 2). Thromboprophylaxis with DOACs was not significantly associated with the risk of MB/CRNMB compared with LMWH/UFH (HR 0.69, CI 95% 0.15–2.16, SHR 0.51, 97.5% CI 0.13–2.03), whereas an ASA score ≥3 was independently associated with a significantly increased bleeding risk (HR 6.02, CI 95% 1.25–28.92, SHR 4.91, 97.5% CI 1.19–20.21), (Table 3). Age was the only variable independently associated with mortality (HR 1.11, CI 95% 1.00–1.22), and thromboprophylaxis with DOACs was not significantly associated with mortality when compared with LMWH/UFH (HR 0.73, CI 95% 0.22–2.37) (Table 4).

**Table 2:** VTE: multivariable Cox and Fine-Gray Regression Analysis.

*Covariate*	Cox Regression		Fine-Gray	
	**HR (95% CI)**	**p-value**	**SHR (97.5% CI)**	**p-value**
***Thromboprophylaxis***				
*LMWH or UFH (ref)*	–	–	–	–
*DOACs*	0.64 (0.16–2.49)	0.517	0.62 (0.16–2.40)	0.490
***Adjustment variables***				
*Prior VTE: No (ref)*	–	–	–	–
*Prior VTE: Yes*	8.87 (2.13–36.98)	0.003	8.89 (2.01–39.35)	**0.004**
*COPD: No (ref)*	–	–	–	–
*COPD: Yes*	3.81 (0.93–15.65)	0.064	3.69 (0.90–15.14)	0.070
Days to surgery*	0.65 (0.40–1.03)	0.069	0.65 (0.41–1.04)	0.072
*Propensity score*	0.99 (0.01–128.70)	0.997	1.11 (0.00–135.88)	0.970

HR = Hazard Ratio; SHR = Subdistribution Hazard Ratio; CI = Confidence Interval; DOACs = Direct Oral Anticoagulants; LMWH = Low Molecular Weight Heparin; UFH = Unfractionated Heparin; VTE = Venous Thromboembolism; COPD = Chronic Obstructive Pulmonary Disease.

* Continuous variables.

**Table 3:** MB/CRNMB: multivariable Cox and Fine-Gray Regression Analysis.

*Covariable*	Cox Regression		Fine-Gray Model	
	HR (95% CI)	p-value	SHR (97.5% CI)	p-value
***Thromboprophylaxis***				
*– LMWH or UFH (ref)*	–	–	–	–
*– DOACs*	0.69 (0.15–2.16)	0.413	0.51 (0.13–2.03)	0.340
***Adjustment variables***				
*ASA score*				
*– ≤ 2 (ref)*	–	–	–	–
*– ≥ 3*	6.02 (1.25–28.92)	0.025	4.91 (1.19–20.21)	**0.027**
*Propensity score*	0.27 (0.00–33.20)	0.595	0.17 (0.00–7.17)	0.350

LMWH = Low Molecular Weight Heparin; UFH = Unfractionated Heparin; DOACs = Direct Oral Anticoagulants; ASA = American Society of Anesthesiologists; HR = Hazard Ratio; SHR = Subdistribution Hazard Ratio; CI = Confidence Interval.

**Table 4:** Mortality: multivariable Cox Regression Analysis.

*Covariable*	Cox Regression	
	HR (95% CI)	p-value
***Thromboprophylaxis***		
*– LMWH or UFH (ref)*	–	–
*– DOACs*	0.73 (0.22–2.37)	0.599
***Adjustment variables***		
*ASA score*		
*– ≤ 2 (ref)*	–	–
*– ≥ 3*	2.16 (0.51–9.12)	0.293
Age*	1.11 (1.00–1.22)	0.030
Creatinine clearance*	0.99 (0.96–1.02)	0.437
*Propensity score*	0.25 (0.00–20.58)	0.535

LMWH = Low Molecular Weight Heparin; UFH = Unfractionated Heparin; DOACs = Direct Oral Anticoagulants; ASA = American Society of Anesthesiologists; HR = Hazard Ratio; CI = Confidence Interval.

* Continuous variables.

## Discussion

This large real-world cohort provides worthy evidence on the comparative effectiveness of DOACs versus LMWH/UFH after hip fracture surgery. Although statistical power was limited by the low number of events, DOACs showed comparable rates of VTE, bleeding, and mortality. These findings are consistent with prior small randomized trials and observational studies suggesting non-inferiority of DOACs compared with LMWH.

We identified seven relevant publications, showing marked heterogeneity in study design, therapeutic strategies, diagnostic criteria, and follow-up. The largest cohort (>3000 patients) was limited to in-hospital events and found no difference between LMWH and DOACs regarding VTE or bleeding [16]. Only two studies extended follow-up beyond 35 days [13,14], while the others assessed outcomes up to 30–35 days postoperatively [10–12,15]. One RCT compared LMWH, DOACs, and sequential therapy (LMWH for 7 days followed by rivaroxaban), finding no significant differences [10]. In contrast, two retrospective studies reported lower VTE with DOACs(14, 15). Reported VTE incidence with DOACs ranged from 0% to 9.5% [10–15], and with LMWH from 1.1% to 26.6% [10–16]. Importantly, most studies included both in-hospital and post-discharge events. In our study, the 3-month post-discharge VTE incidence was 2.9% (2.5% with DOACs vs. 3.9% with LMWH/UFH). Including in-hospital events, the rate increased to 8%, consistent with prior reports.

Bleeding outcomes also showed variability across studies. We observed a 2.5% rate with DOACs and 5.9% with LMWH/UFH, which are slightly lower than most published data, likely explained by exclusion of in-hospital events. Reported rates varied widely, from 0.29% to 15.6% with DOACs and from 0.45% to 11.6% with LMWH [10–13,15]. One RCT with edoxaban found lower bleeding rates with DOACs vs. LMWH (3.4% vs. 6.9%) [11], whereas other studies suggested higher bleeding risk with DOACs, including one with significant differences [15].

Regarding mortality, Lassen et al. reported 0% mortality at 90 days with DOACs vs. 0.9% with LMWH [13]. In our study, mortality was 3.9% with LMWH/UFH and 3% with DOACs. Other studies reported variable 30-day mortality, ranging from 0% [11,15] to 7.4% with DOACs vs. 3.4% with LMWH [12], without significant differences in VTE- or bleeding-related deaths.

Although this was a single-center study with a limited sample, our cohort represents one of the largest reported to date in patients undergoing hip fracture surgery. No statistically significant differences were observed in VTE, MB/CRNMB, or mortality between patients receiving extended prophylaxis with DOACs versus LMWH/UFH. Despite the heterogeneity of published results, our findings in 340 patients are consistent with most prior reports.

This study, however, has several limitations that should be acknowledged. Its retrospective, single-center design entails an inherent risk of information bias and variability in data quality, inconsistency, and imprecision in data quality. In addition, the relatively low number of events resulted in wide confidence intervals in adjusted analyses, limiting the precision of hazard ratio estimates. Potential selection bias may have been introduced, as a larger proportion of patients received DOACs compared with LMWH/UFH. Furthermore, despite propensity score adjustments, some residual imbalance between treatment groups may persist due to unmeasured factors, such as socioeconomic status or temporal confounding. To address these limitations and enhance statistical power, we have established a collaborative project with the Hospital Italiano de Buenos Aires. This ongoing multicenter study will enable the evaluation of a larger cohort, thereby improving estimate precision and providing more robust evidence for the medical community.

## Conclusions

In this retrospective cohort of patients undergoing hip fracture surgery, extended thromboprophylaxis with DOACs showed effectiveness and safety comparable to that of LMWH/UFH. The rates of VTE, MB/CRNMB and mortality were low and showed no statistically significant differences between groups, supporting the notion that DOACs represent a valid alternative for managing this high-risk population. These findings are particularly relevant in real-world practice, where oral administration and cost considerations may favor the use of DOACs.

Nevertheless, the inherent limitations of the retrospective, single-center design and the modest sample size require cautious interpretation of these results. Confirmation through multicenter, prospective studies with greater statistical power is needed to more precisely assess the benefits and risks of DOACs in this setting. The available evidence suggests that, DOACs can be considered an effective and safe option for extended thromboprophylaxis after hip fracture surgery.

## Supporting information

S1 Table Baseline characteristics of patients who developed VTE. (DOCX)

S2 Table Baseline characteristics of patients who experienced bleeding. (DOCX)

S3 Table Baseline characteristics of deceased patients. (DOCX)

S4 Table Clinical events (VTE, bleeding, and mortality) stratified by baseline characteristics. (DOCX)

S1 Data Dataset. (XLSX)
