# Supplementary material for: Extended thromboprophylaxis after hip fracture surgery: Real-world evidence of direct oral anticoagulants versus low molecular weight heparin of unfractionated heparin
Source: PLoS One. 2026 Mar 12;21(3):e0343020. doi: 10.1371/journal.pone.0343020 (PMC12981480; doi:10.1371/journal.pone.0343020)
Supplement: S1 Table — (DOCX) [file pone.0343020.s001.docx]

| Variable | Category | Total (N=10) | LMWH/UFH (N=4) | DOACs (N=6) | p-value |
| --- | --- | --- | --- | --- | --- |
| **Sex (%)** | Female | 10 (100.0) | 4 (100.0) | 6 (100) | NA |
|  | Male | 0 (0.0) | 0 (0.0) | 0 (0.0) |  |
| **Fracture (%)** | Medial | 7 (70.0) | 3 (75.0) | 4 (66.7) | 0.240 |
|  | Lateral | 2 (20.0) | 0 (0.0) | 2 (33.3) |  |
|  | Other/Unknown | 1 (10.0) | 1 (25.0) | 0 (0.0) |  |
| **Surgery (%)** | Total arthroplasty | 1 (10.0) | 0 (0.0) | 89 (37.6) | 0.679 |
|  | Partial arthroplasty | 7 (10.0) | 3 (75.0) | 64 (27.0) |  |
|  | Osteosynthesis | 2 (20.0) | 1 (25.0) | 84 (35.4) |  |
| **Synchronous fracture (%)** | Yes | 0 (0.0) | 0 (0.0) | 0 (0.0) | NA |
|  | No | 10 (100.0) | 4 (100.0) | 6 (100.0) |  |
| **Anesthesia (%)** | General | 1 (10.0) | 0 (0.0) | 1 (16.7) | 0.389 |
|  | Block + sedation | 9 (90.0) | 4 (100.0) | 5 (83.3) |  |
|  | Other | 0 (0.0) | 0 (0.0) | 0 (0.0) |  |
| **ASA score (%)** | <3 | 4 (40.0) | 2 (50.0) | 2 (33.3) | 0.598 |
|  | ≥3 | 6 (60.0) | 2 (50.0) | 4 (66.7) |  |
| **History of VTE (%)** | Yes | 2 (20.0) | 1 (25.0) | 1 (16.7) | 0.747 |
|  | No | 8 (80.0) | 3 (75.0) | 5 (83.3) |  |
| **Known thrombophilia (%)** | Yes | 0 (0.0) | 0 (0.0) | 0 (0.0) | NA |
|  | No | 10 (100.0) | 4 (100.0) | 6 (100.0) |  |
| **COVID-19 during follow-up (%)** | Yes | 0 (0.0) | 0 (0.0) | 0 (0.0) | NA |
|  | No | 10 (100.0) | 4 (100.0) | 6 (100.0) |  |
| **Active cancer (%)** | Yes | 1 (10.0) | 0 (0.0) | 1 (16.7) | 0.389 |
|  | No | 9 (90.0) | 4 (100.0) | 5 (83.3) |  |
| **Smoking (%)** | Active | 2 (20.0) | 1 (25.0) | 1 (16.7) | 0.747 |
|  | No active smoking | 8 (80.0) | 3 (75.0) | 5 (83.3) |  |
| **COPD (%)** | Yes | 3 (30.0) | 2 (50.0) | 1 (16.7) | 0.260 |
|  | No | 7 (70.0) | 2 (50.0) | 5 (83.3) |  |
| **Autoimmune disease (%)** | Yes | 0 (0.0) | 0 (0.0) | 0 (0.0) | NA |
|  | No | 10 (100.0) | 4 (100.0) | 6 (100.0) |  |
| **Hypertension (%)** | Yes | 6 (60.0) | 3 (75.0) | 3 (50.0) | 0.429 |
|  | No | 4 (40.0) | 1 (25.0) | 3 (50.0) |  |
| **Diabetes mellitus (%)** | Yes | 0 (0.0) | 0 (0.0) | 0 (0.0) | NA |
|  | No | 10 (100.0) | 4 (100.0) | 6 (100.0) |  |
| **Dyslipidemia (%)** | Yes | 0 (0.0) | 0 (0.0) | 0 (0.0) | NA |
|  | No | 10 (100.0) | 4 (100.0) | 6 (100.0) |  |
| **Coronary artery disease (%)** | Yes | 1 (10.0) | 0 (0.0) | 1 (16.7) | 0.389 |
|  | No | 9 (90.0) | 4 (100.0) | 5 (83.3) |  |
| **Stroke (%)** | Yes | 0 (0.0) | 0 (0.0) | 0 (0.0) | NA |
|  | No | 10 (100.0) | 4 (100.0) | 6 (100.0) |  |
| **Peripheral artery disease (%)** | Yes | 1 (10.0) | 0 (0) | 1 (16.7) | 0.389 |
|  | No | 9 (90.0) | 4 (100.0) | 5 (83.3) |  |
| **Period (%)** | 2011–2017 | 4 (40.0) | 4 (100.0) | 0 (0.0) | 0.002 |
|  | 2018–2025 | 6 (60.0) | 0 (0.0) | 6 (100.0) |  |
| **Age: median (IQR)** | – | 84.35 (81.74–86.18) | 84.35 (83.10–85.41) | 83.79 (81.74–90.29) | 0.747 |
| **Surgery time (min), mean (SD)** | – | 88.20 (32.29) | 93.75 (43.08) | 84.50 (26.66) | 0.719 |
| **Creatinine clearance, median (IQR)** | – | 80.50 (74.75–86.50) | 82.50 (79.25–86.0) | 77.50 (70.25–85.50) | 0.476 |
| **Days to surgery, median (IQR)** | – | 1 (1–2.75) | 1 (1–1.5) | 1.5 (1–3.5) | 0.470 |
| **Hospital stay (days), median (IQR)** | – | 9 (6–10) | 9.5 (8–11) | 7.5 (6–9.75) | 0.666 |
| **BMI, median (IQR)** | – | 21.91 (19.53–22.94) | 22.94 (20.78–23.73) | 21.65 (20.00–22.58) | 0.905 |

LMWH = Low Molecular Weight Heparin; UFH = Unfractionated Heparin; DOACs = Direct Oral Anticoagulants; VTE = Venous Thromboembolism; COPD = Chronic Obstructive Pulmonary Disease; ASA = American Society of Anesthesiologists; BMI = Body Mass Index; SD = Standard Deviation; IQR = Interquartile Range.
