# Supplementary material for: Extended thromboprophylaxis after hip fracture surgery: Real-world evidence of direct oral anticoagulants versus low molecular weight heparin of unfractionated heparin
Source: PLoS One. 2026 Mar 12;21(3):e0343020. doi: 10.1371/journal.pone.0343020 (PMC12981480; doi:10.1371/journal.pone.0343020)
Supplement: S2 Table — (DOCX) [file pone.0343020.s002.docx]

| Variable | Category | Total  (N=12) | LMWH/UFH (N=6) | DOACs (N=6) | p-value |
| --- | --- | --- | --- | --- | --- |
| **Sex (%)** | Female | 9 (81.8) | 5 (83.3) | 5 (83.3) | 1 |
|  | Male | 2 (18.2) | 1 (16.7) | 1 (16.7) |  |
| **Fracture (%)** | Medial | 4 (33.3) | 2 (33.3) | 2 (33.3) | 0.091 |
|  | Lateral | 5 (41.7) | 1 (16.7) | 4 (66.7) |  |
|  | Other/Unknown | 3 (25.0) | 3 (50.0) | 0 (0.0) |  |
| **Surgery (%)** | Total arthroplasty | 5 (41.7) | 3 (50.0) | 2 (33.3) | 0.819 |
|  | Partial arthroplasty | 2 (16.7) | 1 (16.7) | 1 (16.7) |  |
|  | Osteosynthesis | 5 (41.7) | 2 (33.3) | 3 (50.0) |  |
| **Synchronous fracture (%)** | Yes | 1 (8.3) | 1 (16.7) | 0 (0.0) | 0.338 |
|  | No | 11 (91.7) | 5 (83.3) | 6 (100.0) |  |
| **Anesthesia (%)** | General | 4 (33.3) | 2 (33.3) | 2 (33.3) | 0.497 |
|  | Block + sedation | 7 (58.3) | 3 (50.0) | 4 (66.7) |  |
|  | Other | 1 (8.3) | 1 (16.7) | 0 (0.0) |  |
| **ASA score (%)** | <3 | 4 (33.3) | 3 (50.0) | 1 (16.7) | 0.221 |
|  | ≥3 | 8 (66.7) | 3 (50.0) | 5 (83.3) |  |
| **History of VTE (%)** | Yes | 0 (0.0) | 0 (0.0) | 0 (0.0) | NA |
|  | No | 12 (100.0) | 6 (100.0) | 6 (100.0) |  |
| **Known thrombophilia (%)** | Yes | 0 (0.0) | 0 (0.0) | 0 (0.0) | NA |
|  | No | 12 (100.0) | 6 (100.0) | 6 (100.0) |  |
| **COVID-19 during follow-up (%)** | Yes | 0 (0.0) | 0 (0.0) | 0 (0.0) | NA |
|  | No | 12 (100.0) | 6 (100.0) | 6 (100.0) |  |
| **Active cancer (%)** | Yes | 1 (8.3) | 0 (0.0) | 1 (16.7) | 0.296 |
|  | No | 11 (91.7) | 6 (100.0) | 5 (83.3) |  |
| **Smoking (%)** | Active | 5 (41.7) | 4 (66.7) | 1 (16.7) | 0.079 |
|  | No active smoking | 6 (58.3) | 2 (33.3) | 4 (83.3) |  |
| **COPD (%)** | Yes | 3 (25.0) | 2 (33.3) | 1 (16.7) | 0.505 |
|  | No | 9 (75.0) | 4 (66.7) | 4 (83.3) |  |
| **Autoimmune disease (%)** | Yes | 0 (0.0) | 0 (0.0) | 0 (0.0) | NA |
|  | No | 12 (100.0) | 6 (100.0) | 6 (100.0) |  |
| **Hypertension (%)** | Yes | 8 (66.7) | 4 (66.7) | 4 (66.7) | 1 |
|  | No | 4 (33.3) | 2 (33.3) | 2 (33.3) |  |
| **Diabetes mellitus (%)** | Yes | 1 (8.3) | 0 (0.0) | 1 (16.7) | 0.296 |
|  | No | 11 (91.7) | 6 (100.0) | 4 (83.3) |  |
| **Dyslipidemia (%)** | Yes | 2 (16.7) | 2 (33.3) | 0 (0.0) | 0.121 |
|  | No | 10 (83.3) | 4 (66.7) | 6 (100.0) |  |
| **Coronary artery disease (%)** | Yes | 2 (16.7) | 2 (33.3) | 0 (0.0) | 0.121 |
|  | No | 10 (83.3) | 4 (66.7) | 6 (100.0) |  |
| **Stroke (%)** | Yes | 4 (33.3) | 2 (33.3) | 2 (33.3) | 1 |
|  | No | 8 (66.7) | 4 (66.7) | 4 (66.7) |  |
| **Peripheral artery disease (%)** | Yes | 3 (25.0) | 2 (33.3) | 1 (16.7) | 0.505 |
|  | No | 9 (75.0) | 4 (66.7) | 5 (83.3) |  |
| **Period (%)** | 2011–2017 | 7 (58.3) | 6 (100.0) | 1 (16.7) | 0.003 |
|  | 2018–2025 | 5 (41.7) | 0 (0.0) | 5 (83.3) |  |
| **Age: median (IQR)** | – | 83.06 (78.25–87.82) | 81.17 (79.47–83.40) | 85.41 (76.78–89.16) | 0.699 |
| **Surgery time (min), median (IQR)** | – | 88.00 (63.00-114.75) | 98.5 (69.75-117.50) | 75.5 (61.0-107.0) | 0.688 |
| **Creatinine clearance, median (IQR)** | – | 68.15 (39.65–80.75) | 78.00 (63.25.–82.25) | 50.25 (38.55–74.32) | 0.484 |
| **Days to surgery, median (IQR)** | – | 3.00 (2.75–4.00) | 3.00 (3.00–3.00) | 4.00 (250–4.00) | 0.454 |
| **Hospital stay (days), median (IQR)** | – | 10.00 (8.00–11.5) | 11 (10.25–14.75) | 8.00 (6.50–8.75) | 0.030 |
| **BMI, median (IQR)** | – | 20.08 (19.53–27.24) | 22.93 (20.78–25.09) | 25.08 (22.30–30.90) | 0.800 |

LMWH = Low Molecular Weight Heparin; UFH = Unfractionated Heparin; DOACs = Direct Oral Anticoagulants; VTE = Venous Thromboembolism; COPD = Chronic Obstructive Pulmonary Disease; ASA = American Society of Anesthesiologists; BMI = Body Mass Index; SD = Standard Deviation; IQR = Interquartile Range.
