# Supplementary material for: Extended thromboprophylaxis after hip fracture surgery: Real-world evidence of direct oral anticoagulants versus low molecular weight heparin of unfractionated heparin
Source: PLoS One. 2026 Mar 12;21(3):e0343020. doi: 10.1371/journal.pone.0343020 (PMC12981480; doi:10.1371/journal.pone.0343020)
Supplement: S3 Table — (DOCX) [file pone.0343020.s003.docx]

| Variable | Category | Total  (N=11) | LMWH/UFH (N=4) | DOACs (N=7) | p-value |
| --- | --- | --- | --- | --- | --- |
| **Sex (%)** | Female | 8 (72.7) | 2 (50.0) | 6 (85.7) | 0.201 |
|  | Male | 3 (27.3) | 2 (50.0) | 1 (14.3) |  |
| **Fracture (%)** | Medial | 4 (36.4) | 1 (25.0) | 3 (42.9) | 0.021 |
|  | Lateral | 4 (36.4) | 0 (0.0) | 4 (57.1) |  |
|  | Other/Unknown | 3 (27.3) | 3 (75.0) | 0 (0.0) |  |
| **Surgery (%)** | Total arthroplasty | 2 (18.2) | 2 (50.0) | 0 (0.0) | 0.055 |
|  | Partial arthroplasty | 5 (45.5) | 2 (50.0) | 3 (42.9) |  |
|  | Osteosynthesis | 4 (36.4) | 0 (0.0) | 4 (57.1) |  |
| **Synchronous fracture (%)** | Yes | 2 (18.2) | 1 (25.0) | 1 (14.3) | 0.296 |
|  | No | 9 (81.8) | 3 (75.0) | 6 (85.7) |  |
| **Anesthesia (%)** | General | 3 (27.3) | 0 (0.0) | 3 (42.9) | 0.125 |
|  | Block + sedation | 8 (72.7) | 4 (100.0) | 4 (57.1) |  |
|  | Other | 0 (0.0) | 0 (0.0) | 0 (0.0) |  |
| **ASA score (%)** | <3 | 3 (27.3) | 1 (25.0) | 2 (28.6) | 0.898 |
|  | ≥3 | 8 (72.7) | 3 (75.0) | 5 (71.4) |  |
| **History of VTE (%)** | Yes | 0 (0.0) | 0 (0.0) | 0 (0.0) | NA |
|  | No | 12 (100.0) | 4 (100.0) | 7 (100.0) |  |
| **Known thrombophilia (%)** | Yes | 0 (0.0) | 0 (0.0) | 0 (0.0) | NA |
|  | No | 12 (100.0) | 4 (100.0) | 7 (100.0) |  |
| **COVID-19 during follow-up (%)** | Yes | 1 (9.1) | 0 (0.0) | 1 (14.3) | 0.428 |
|  | No | 10 (90.9) | 4 (100.0) | 6 (85.7) |  |
| **Active cancer (%)** | Yes | 2 (18.2) | 1 (25.0) | 1 (14.3) | 0.658 |
|  | No | 9 (81.8) | 3 (75.0) | 6 (85.7) |  |
| **Smoking (%)** | Active | 3 (27.3) | 2 (50.0) | 1 (14.3) | 0.201 |
|  | No active smoking | 8 (72.7) | 2 (50.0) | 6 (85.7) |  |
| **COPD (%)** | Yes | 3 (27.3) | 2 (50.0) | 1 (14.3) | 0.201 |
|  | No | 8 (72.7) | 2 (50.0) | 6 (85.7) |  |
| **Autoimmune disease (%)** | Yes | 0 (0.0) | 0 (0.0) | 0 (0.0) | NA |
|  | No | 12 (100.0) | 4 (100.0) | 7 (100.0) |  |
| **Hypertension (%)** | Yes | 9 (81.8) | 4 (100.0) | 5 (71.4) | 0.237 |
|  | No | 2 (18.2) | 0 (0.0) | 2 (28.6) |  |
| **Diabetes mellitus (%)** | Yes | 2 (18.2) | 1 (25.0) | 1 (14.3) | 0.658 |
|  | No | 9 (81.8) | 3 (75.0) | 6 (85.7) |  |
| **Dyslipidemia (%)** | Yes | 3 (27.3) | 2 (50.0) | 1 (14.3) | 0.201 |
|  | No | 8 (72.7) | 2 (50.0) | 6 (85.7) |  |
| **Coronary artery disease (%)** | Yes | 1 (9.1) | 1 (25.0) | 0 (0.0) | 0.165 |
|  | No | 10 (90.9) | 3 (75.0) | 7 (100.0) |  |
| **Stroke (%)** | Yes | 3 (27.3) | 2 (33.3) | 1 (14.3) | 0.201 |
|  | No | 8 (72.7) | 2 (50.0) | 6 (85.7) |  |
| **Peripheral artery disease (%)** | Yes | 2 (18.2) | 2 (50.0) | 0 (0.0) | 0.039 |
|  | No | 9 (81.8) | 2 (50.0) | 7 (100.0) |  |
| **Period (%)** | 2011–2017 | 3 (27.3) | 3 (75.0) | 0 (00.0) | 0.007 |
|  | 2018–2025 | 8 (72.7) | 1 (25.0) | 7 (100.0) |  |
| **Age: median (IQR)** | – | 88.35 (87.33–91.65) | 84.86 (81.07–89.00) | 90.96 (88.22–92.31) | 0.163 |
| **Surgery time (min), median (IQR)** | – | 90.00 (49.00-102) | 100.00 (78.75-112.50) | 54.00 (47.0-93.0) | 0.527 |
| **Creatinine clearance, median (IQR)** | – | 59.00 (37.50–76.50) | 62.00 (51.50.–68.00) | 52.0 (37.5–80.00) | 0.927 |
| **Days to surgery, median (IQR)** | – | 2.00 (1.50–6.00) | 1.50 (1.00–3.00) | 6.00 (2.00–6.50) | 0.289 |
| **Hospital stay (days), median (IQR)** | – | 12.00 (8.00–17.5) | 10.5 (8.75–13.00) | 13.00 (9.00–21.50) | 0.570 |
| **BMI, median (IQR)** | – | 22.94 (20.97–23.02) | 22.94 (22.94–22.94) | 21.05 (20.03–22.08) | 1 |

LMWH = Low Molecular Weight Heparin; UFH = Unfractionated Heparin; DOACs = Direct Oral Anticoagulants; VTE = Venous Thromboembolism; COPD = Chronic Obstructive Pulmonary Disease; ASA = American Society of Anesthesiologists; BMI = Body Mass Index; SD = Standard Deviation; IQR = Interquartile Range.
