# Supplementary material for: Extended thromboprophylaxis after hip fracture surgery: Real-world evidence of direct oral anticoagulants versus low molecular weight heparin of unfractionated heparin
Source: PLoS One. 2026 Mar 12;21(3):e0343020. doi: 10.1371/journal.pone.0343020 (PMC12981480; doi:10.1371/journal.pone.0343020)
Supplement: S4 Table — (DOCX) [file pone.0343020.s004.docx]

| Variable | Category | TVE  N=10 | MB or NMCRB  N=12 | Deaths  N=11 |
| --- | --- | --- | --- | --- |
| **Age** | <75 | 1/69 | 3/69 | 0/69 |
|  | 75-85 | 4/127 | 5/127 | 2/127 |
|  | ≥ 85 | 5/144 | 4/144 | 9/144 |
| **Creatinine clearance** | ≥ 60 mL/min | 9/231 | 7/231 | 5/231 |
|  | 30-59 mL/min | 1/95 | 5/95 | 5/95 |
|  | < 30 mL/min | 0/10 | 0/10 | 1/10 |
| **Fracture** | Medial | 7/207 | 4/207 | 4/207 |
|  | Lateral | 2/98 | 5/98 | 4/98 |
|  | Other/Unknown | 1/35 | 3/35 | 3/35 |
| **ASA score** | <3 | 4/195 | 4/195 | 3/195 |
|  | ≥3 | 6/143 | 8/143 | 8/143 |
| **Surgery (%)** | Total arthroplasty | 1/144 | 5/144 | 2/144 |
|  | Partial arthroplasty | 7/90 | 2/90 | 5/90 |
|  | Osteosynthesis | 2/105 | 5/105 | 4/105 |

ASA = American Society of Anesthesiologists
